# Parameter Identification of an Ultrafiltration Model for Organics Removal in a Full-Scale Wastewater Reclamation Plant with Sparse and Incomplete Monitoring Data

**DOI:** 10.1371/journal.pone.0161300

**Published:** 2016-08-16

**Authors:** Fu Sun, Siyu Zeng, Yunqing Huang, Miao He

**Affiliations:** State Key Joint Laboratory of Environment Simulation and Pollution Control, School of Environment, Tsinghua University, Beijing, 100084, China; UCLA, UNITED STATES

## Abstract

Ultrafiltration (UF) has become one of the dominant treatment processes for wastewater reclamation in China. Modeling is an effective instrument to understand and optimize UF systems. To this end, a previously developed UF model for organics removal was applied to the UF process in a typical, full-scale wastewater reclamation plant (WRP) in China. However, the sparse and incomplete field monitoring data from the studied WRP made the traditional model analysis approaches hardly work in this case. Therefore, two strategies, namely Strategy 1 and Strategy 2, were proposed, following a regional sensitivity analysis approach, for model parameter identification. Strategy 1 aimed to identify the model parameters and the missing model input, i.e. sampling times, simultaneously, while Strategy 2 tried to separate these two processes to reduce the dimension of the identification problem through an iteration procedure. With these two strategies, the model performed well in the Qinghe WRP with the absolute relative errors between the simulated and observed total organic carbon (TOC) generally below 10%. The four model parameters were all sensitive and identifiable, and even the sampling times could be roughly identified. Given the incomplete model input, these results were encouraging and added to the trustworthiness of model when it was applied to the Qinghe WRP.

## Introduction

Ultrafiltration (UF) is a membrane filtration technology used for the separation of macromolecular solids from liquid. UF has been widely used in food, chemical and pharmaceutical industries since its development in the 1960s, and its application to water and wastewater treatment has been accelerated in the recent 30 years [1,2]. The advantages of UF technology lie in its ease of operation, high quality of permeate, and small space requirement [3].

Modeling UF process is critical to understanding, operating and optimizing UF systems, and various models have been developed to describe UF process. However, most of them have focused on the flux behavior of UF [4,5], and less attention has been paid to water quality. Moreover, the existing water quality models for UF have been developed specifically for food, chemical and pharmaceutical industries [6–8], including theoretical models based on concentration polarization phenomenon [9,10], hindered transport theory [11,12], and pore blocking effect [13,14], as well as empirical models based on multiple regression analysis [7,15] and artificial neutral network [6,16]. These models were mainly developed for and applied to single-solute systems. Therefore, their applicability to UF in water and wastewater treatment processes, where the components of the solution are complicated and usually not fully understood, is subject to scrutiny.

With the rapid expansion of wastewater reuse in Beijing, China, UF has become a dominant process in the wastewater treatment chain. Aiming at better understanding and optimizing the UF performance in organics rejection in wastewater reclamation, a water quality model has been developed and validated against data from pilot-scale experiments in a previous study [17]. This paper will present a preliminary effort to test the applicability of this model to the UF process in a full-scale wastewater reclamation plant (WRP) with historical monitoring data. What makes this model test study complicated, however, is that the available monitoring data from the WRP was sparse and incomplete, and more specifically, the sampling times for the water quality data were not recorded. With such important input missing, two strategies based on regional sensitivity analysis (RSA) were designed for model parameter identification, and both the goodness-of-fit of the model and the sensitivity, identifiability, and uncertainty of the model parameters were examined in this paper. In the next section, the previously developed model, the WRP and its monitoring data were briefly introduced, and then two strategies for model parameter identification were presented. Following that, the model performance as well as the identified model parameters and sampling times was analyzed. Finally, the difference between the two strategies was discussed before concluding the paper.

## Methods

### The UF Model for Organics Removal in Wastewater Reclamation

The model assumes that organics rejection by UF is controlled by the combined effects of concentration polarization and pore blocking processes. On the one hand, the rejected solute accumulating on the membrane surface will increase the thickness and resistance of the boundary layer and thus affect solute transport and permeate flux. On the other hand, the movement of solute molecules across the membrane will be affected by the reduced effective area as a result of pore blocking in the membrane. Assuming a constant flux, the permeate organics of the UF process could be described by Eq (1).

$$\frac{A_{0}}{lQ}f\times (c_{\text{b}}-c_{\text{p}})\times (1-at\text{+}bt^{2}-ct^{3})=t\frac{\text{d}c_{\text{p}}}{\text{d}t}+c_{\text{p}}$$ (1)

In Eq (1), *A*_0_ refers to the initial effective area of the membrane (m^2^), *l* denotes the thickness of the membrane (m), *Q* indicates the flow rate (m^3^/s), *c*_b_ and *c*_p_ represent the feed and permeate concentration respectively (kg/m^3^), *t* is time (s), and *a*, *b*, *c* and *f* are the model parameters. Among the four model parameters, *a*, *b* and *c* characterize the pore blocking rate, while *f* is an aggregated parameter incorporating the influences of solute characteristics, hydraulic status and operating conditions. Details about model development can be found in [17].

Data from a series of pilot-scale experiments (PSEs), where organics were characterized by ultraviolet absorbance at 254 nm (UV_254_), were used for model parameter identification and structure validation following the model analysis methodology developed by [18], and details were described elsewhere [17]. It was found that the model, on the one hand, could well predict the permeate UV_254_ with absolute relative errors less than 10% for most observed data, and on the other hand, had a balanced structure with all the four parameters well identified and their uncertainties significantly reduced [17]. Therefore, the developed model exhibited a robust and reliable structure and would be assumedly applicable to other similar situation.

### The WRP and Data

In this study, the previously developed model was applied to a full-scale WRP, namely the Qinghe WRP in Beijing, China (116°22’5” E, 40°2’53” N). The WRP employs a conventional activated sludge process, coupled with subsequent UF and ozone oxidation processes, and its effluent is used for multiple purposes, such as urban stream augmentation, industrial cooling systems, toilet flushing in households, and landscape irrigation. The UF system consists of six trains of ZeeWeed-1000 hollow-fiber membranes, and each train contains nine cassettes with 57 or 60 membrane modules per cassette. The membrane is made of polyvinylidene fluoride and has a nominal pore size of 0.02 μm. The UF system is operated in an outside-in mode at a constant flow up to 23 L (m^2^ h)^-1^, and the net production capacity reaches 80,000 m^3^ d^-1^. Each cassette is hydraulically backwashed 29 times per day and chemically maintained once every day.

From the water quality perspective, organics have the potential to increase the biological instability, toxicity, and aesthetical unacceptability of the reclaimed wastewater, and thus their concentration, usually characterized by an aggregated index, e.g. chemical oxygen demand (COD), biological oxygen demand (BOD) and total organic carbon (TOC), has been regulated by the water quality standards for both wastewater discharge into recipient water and reclaimed wastewater for various uses in China. Furthermore, Beijing released a more stringent water quality standard for the discharge from municipal wastewater treatment plants into recipient water in 2012, which is relevant to wastewater reuse for stream and lake augmentation. According to this standard, the allowed concentration for TOC of the treated water from the Qinghe WRP should not be higher than 12 mg L^-1^, while those for COD and BOD should decrease from 60 mg L^-1^ to 30 mg L^-1^ and from 20 mg L^-1^ to 6 mg L^-1^, respectively, since 31 December 2015. To this end, the studied WRP needs to evaluate the efficiency of its treatment processes in removing organics, understand its influencing factors and thus explore its potential to enhance removal.

This study specifically focuses on the UF system of the Qinghe WRP, and historical monitoring data was obtained to examine the organics rejection performance of the UF system. As part of a joint research project, the monitoring program was approved by the Beijing Drainage Group Co., LTD and conducted between from June 2012 and March 2013 on a monthly basis. Water samples were collected from both feed and permeate of the UF system, and their TOC concentrations were tested. The feed TOC concentrations varied between 5.25 mg L^-1^ and 18.65 mg L^-1^, while those of the permeate ranged from 5.03 mg L^-1^ and 17.31 mg L^-1^. TOC removal rate was found to vary between -20.6% and 33.2%, with an average of 9.5%.

### Application of the UF Model to the Qinghe WRP

As already mentioned, this study aimed to examine the applicability of the previously developed UF model, which had been validated against data from the PSEs, to the full-scale Qinghe WRP. However, since the existing monitoring program was not specifically designed for modeling the UF system, the available historical data as described previously brings about two major challenges to this model test study. Firstly, the water quality indicator used to represent organics in the UF system of the WRP was TOC, which was different from UV_254_ used in the PSEs. So the model parameters needed to be recalibrated when the model structure remained unchanged, assuming that TOC and UV_254_ were equivalent indicators of organics and behaved similarly during the UF process. The second and more critical challenge was that the monitoring data available was sparse and incomplete for model development and validation. On the one hand, water samples from the UF system of the WRP were collected on a monthly basis from separate operation cycles with different feed TOC concentrations, which was too sparse as compared with the PSEs in which water samples were collected consecutively in a whole operation cycle under well-controlled conditions. On the other hand, the sampling times of the water samples in the WRP were not recorded, which had defeated the authors’ effort to derive the corresponding sampling moments in their operation cycles based on the data, e.g. transmembrane pressure, from the online monitoring system.

Data sparsity is not uncommon in environmental modeling studies. This could result from lack of appropriate monitoring instruments or capacity as well as inadequate temporal/spatial resolution, poor representativeness or low accuracy of the monitoring instruments [19–22]. There are also cases where model parameters or input are difficult to monitor, e.g. diffuse pollution loads [23], or even unknown, e.g. sources of groundwater pollution [24–26]. Different methods have been developed and applied to deal with data sparsity issues in modeling studies. A straightforward method is to use other theoretical or empirical methods to estimate the missing model parameters or input. For example, Nyeko [22] estimated the missing records of solar radiation for hydrological modeling with an empirical equation. Inverse modeling is another widely applied method to determine missing model input. For example, Hörmann et al. [27] estimated the fraction of wetland in a catchment in inverse modeling runs of a hydrological model, while Herrnegger et al. [21] used inverse rainfall-runoff modeling to obtain additional information on mean areal rainfall of the studied area. Inverse modeling, however, generally requires the model be calibrated. If model parameters and input are both unknown or partially known yet with uncertainty, simultaneous identification of parameters and input is normally applied. For example, due to the difficulty in detecting groundwater pollution sources, many researchers such as [24–26] developed methods to identify source characteristics (e.g. location, magnitude, duration) and at the same time estimate unknown aquifer parameters. Similarly, Jun et al. [23] used an optimal algorithm in river water quality modeling for simultaneous estimation of kinetic constants and diffuse loads of total nitrogen and phosphorus. In order to reduce uncertainty resulting from precipitation observations and parameter estimation in hydrological modeling, Pluntke et al. [20] used an ensemble approach by establishing a series of models, among which a model allowed the precipitation input to be calibrated together with other model parameters.

**Fig 1:**
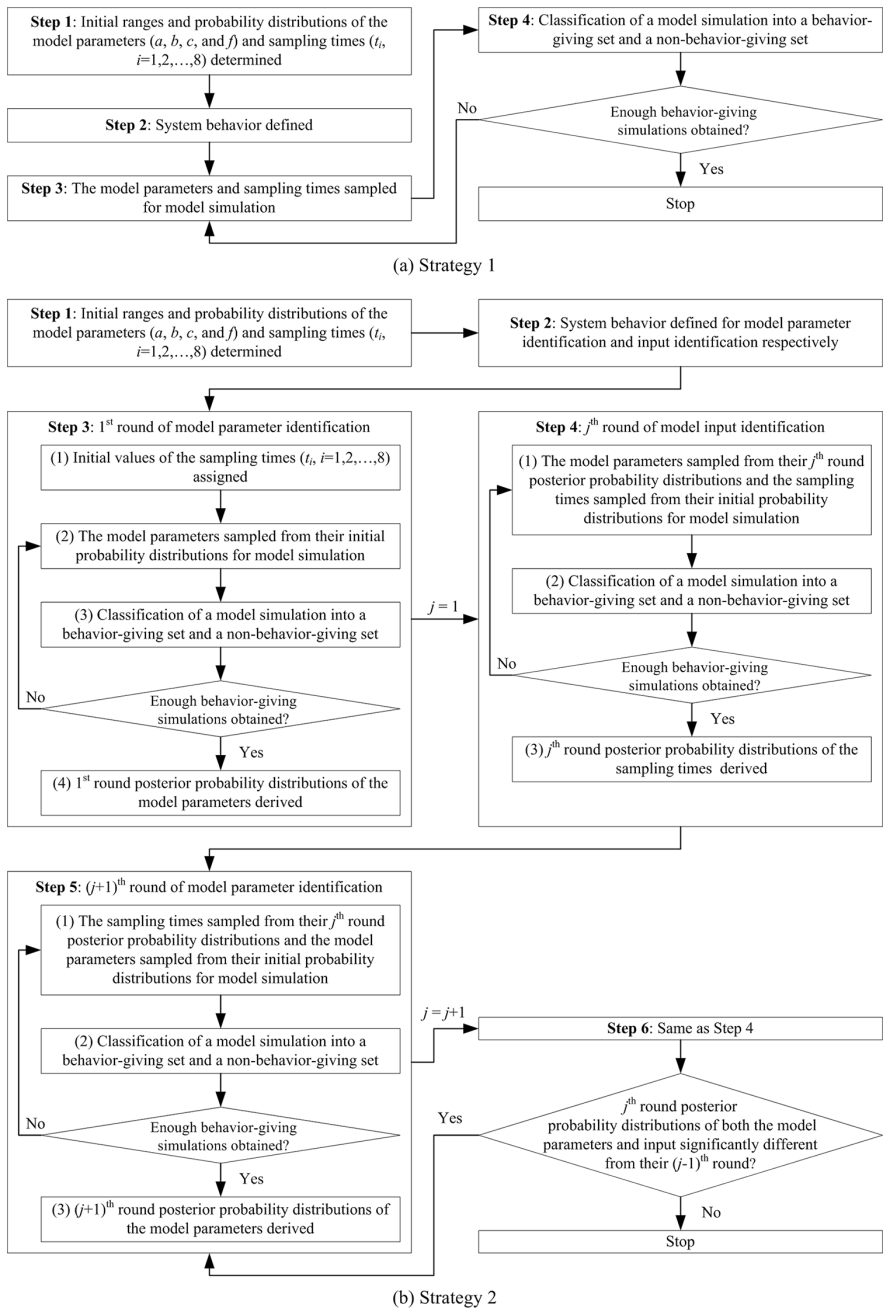
Two strategies for parameter identification of the UF model applied to the Qinghe WRP.

Since UF systems operate in cycles in the Qinghe WRP, sampling times are obviously critical model input. In light of the aforementioned existing approaches to dealing with data sparsity, simultaneous identification of model parameters and sampling times could be the only feasible choice in this case. Before proceeding to detailed algorithm design, another assumption has to be made that the UF process in the WRP operated in a cyclic yet steady state, which could be justified given that no abnormality was reported during the monitoring period. With this assumption, two strategies for model identification were proposed as shown in Fig 1 and details are given below. A common feature of these two strategies is that they are both based on the RSA approach to consider the gross uncertainty associated with model parameters and sampling times. Furthermore, RSA was both performed with the Hornberger-Spear-Young (HSY) algorithm, the procedure of which has been detailed in [18] and [28], based on a Latin Hypercube Sampling (LHS) approach.

Similar to the current practice of simultaneous identification of model parameters and input, e.g. [20] and [23], Strategy 1 regarded the missing model input, i.e. the sampling times of the observed data (*t*_*i*_, *i* = 1,2,…,8), as “parameters” that needed to be identified together with the four model parameters, i.e. *a*, *b*, *c* and *f*. Following the procedure of the HSY algorithm, as shown in Fig 1(a), the initial ranges of the model parameters and sampling times were first determined, as shown in Table 1, according to the reported values in literature (see [17]) and the operating conditions in the Qinghe WRP respectively. For simplicity, uniform distributions were assumed for all model parameters and sampling times. Then system behavior was defined to set a criterion for a model simulation to be accepted as a behavior-giving one. Herein a behavior-giving simulation was defined as one with at least 60% of the data points that had absolute relative errors less than 20% between the observed and simulated *c*_p_. In the third step, both the model parameters and the sampling times were randomly and independently sampled with a LHS approach according to their designated ranges and probability distributions, and then these values were substituted into Eq (1) for model simulation. The simulation results, together with the parameters and sampling times, were later classified into a behavior-giving set and a non-behavior-giving set according to the definition of system behavior. The simulation continued, i.e. Step 3 and Step 4 in Fig 1(a), until enough behavior-giving simulations were obtained, and finally posterior probability distributions (PPDs) of the parameters and sampling times could be derived for further analysis.

**Table 1:** Initial ranges and probability distributions of model parameters and input.

Parameter/input	Unit	Initial range	Prior probability distribution
*a*	-	(0, 1.0×10^−3^)	Uniform
*b*	-	(0, 5.0×10^−6^)	Uniform
*c*	-	(0, 1.0×10^−9^)	Uniform
*f*	-	(0, 7.0×10^−8^)	Uniform
*t*_*i*_ (*i* = 1,2,…,8)	min	(0, 50)	Uniform

Strategy 1 calibrated the four model parameters and eight sampling times simultaneously, which actually increased the dimension of the identification problem, and therefore great uncertainty were expected to remain with these parameters and input. Given that the four model parameters had global impact on all the eight observations while the sample times only had local impact on each specific observation, it is possible to reduce the dimension of the identification problem temporarily by separating the identification of model parameters from that of sampling times at different stages. Based on this idea, Strategy 2 was designed as shown in Fig 1(b). In the first step, the same initial ranges and probability distributions as those in Strategy 1 were assigned to the four model parameters and eight sampling times. Then the definition of system behavior in Strategy 1 was also adopted at the stage of model parameter identification. At the stage of model input identification, however, a little stricter definition of system behavior was applied and a behavior-giving simulation was one with at least 60% of the data points that had absolute relative errors less than 15% between the observed and simulated *c*_p_. The first round of model parameter identification followed and started with randomly and independently sampling the eight sampling times, only once, according to their designated ranges and probability distributions shown in Table 1. These values were later fixed in this round of simulation as if they were known model input, while the four model parameters were calibrated following the same HSY algorithm as that of Strategy 1. At the end of this round of simulation, the PPDs of the four model parameters could be derived. This step was followed by the first round of model input identification where the four model parameters were assumed to have known probability distributions, i.e. their first round PPDs, while the eight sampling times were calibrated also following same HSY algorithm. Since the eight observations were independently obtained from different operation cycles of the UF system, the calibration of eight sampling times could be done individually. That is to say there is only one unknown sampling time to be calibrated for each observation. Similarly, this round of simulation would end up with the PPDs of eight sampling times, which were later used for the second round of model parameter identification as shown in Fig 1(b). When the second round PPDs of both model parameters and sampling times were obtained, they were compared with their first round PPDs through the Kolmogorov-Smirnov (K-S) test, at a 0.05 significance level, to examine whether the PPD for each parameter and sampling time was significantly different between these two rounds of simulation. If any parameter or sampling time showed a significant difference, another round of model parameter identification and input identification would be conducted, i.e. Step 5 and Step 6 in Fig 1(b), and the latest derived PPDs could then be compared with the previous ones to examine convergence for all these model parameters and input. The iterations terminated when no statistically significant differences were detected in the PPDs for all the model parameters and sampling times between two recent iterations.

The two strategies were then compared with respect to their model performance, and the sensitivity, identifiability, and uncertainty of the model parameters as well as the missing model input, i.e. sampling times of the observed data. Model performance was evaluated by the absolute relative errors between the observed and simulated data. Sensitivity, identifiability, and uncertainty of model parameters could be used to judge a model’s reliability [18]. A model with a large proportion of sensitive parameters will have a balanced model structure, and the model will be more trustworthy when the sensitive parameters could be well identified with low uncertainty. In this study, regional sensitivity for each model parameter was characterized by the statistical difference in the two PPDs between the behavior-giving set and the non-behavior-giving set through the K-S test at a 0.05 significance level. The greater the difference between the two distributions, the more sensitive and identifiable the parameter is. Furthermore, the standard deviation of the behavior-giving set was used as an indicator of uncertainty for each model parameter. A smaller standard deviation of a parameter usually indicates better identifiability and lower uncertainty. Since the sampling times were also identified in this study, the sensitivity, identifiability, and uncertainty could be evaluated similarly.

## Results and Discussion

### Model Performance

**Fig 2:**
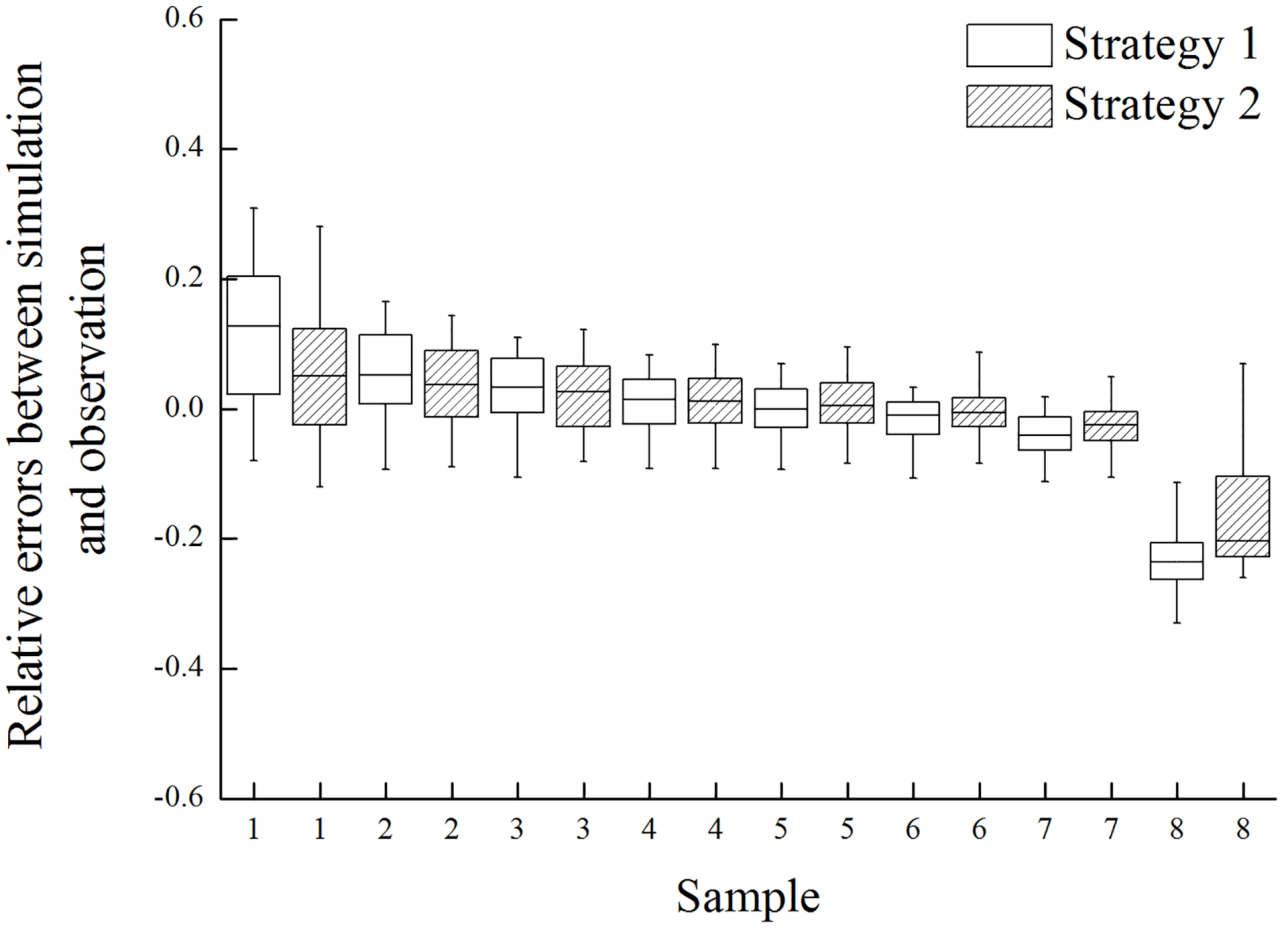
Relative errors between the simulated and observed permeate TOC with Strategy 1 and 2.

Fig 2 shows the relative errors between the simulated and observed permeate TOC of the UF process in the Qinghe WRP from the 100 best behavior-giving simulations for both Strategy 1 and Strategy 2 in box-and-whisker plots. In the figure, the lower and upper boundaries of the box represented the first and the third quartiles of the relative errors respectively, while the line inside the box marked the median. Outside the box, two vertical whiskers extended downwards to the 5th percentile and upwards to the 95th percentile respectively. As shown in Fig 2, the absolute relative errors between the simulated and observed TOC were generally below 10% for Sample 1~7 with both Strategy 1 and Strategy 2, while those for sample 8 were the greatest. A possible reason could be that Sample 8 was the only one that had a negative TOC rejection rate of -20.6%, which would be regarded as a rare event for the UF model to simulate based on the hypothetical mechanisms. Overall speaking, the previously developed UF model could provide good simulation performance for the Qinghe WRP, even though TOC was herein used instead of UV_254_ in the PSEs dataset and the sampling times of water samples were missing.

It could also be observed from Fig 2 that the absolute relative errors for Sample 1, 2, and 8 were greater than other samples. In these three cases, Strategy 2 performed a little better than Strategy 1 possibly due to a stricter definition of system behavior at the stage of model input identification in Strategy 2. In addition, separate identification of the sampling time for each water sample at this stage in Strategy 2 may also be part of the reason for its good performance in these cases. So Strategy 2 was a more robust option for model parameter identification in the case of the Qinghe WRP especially when the model input was incomplete.

### Identified Model Parameters

**Fig 3:**
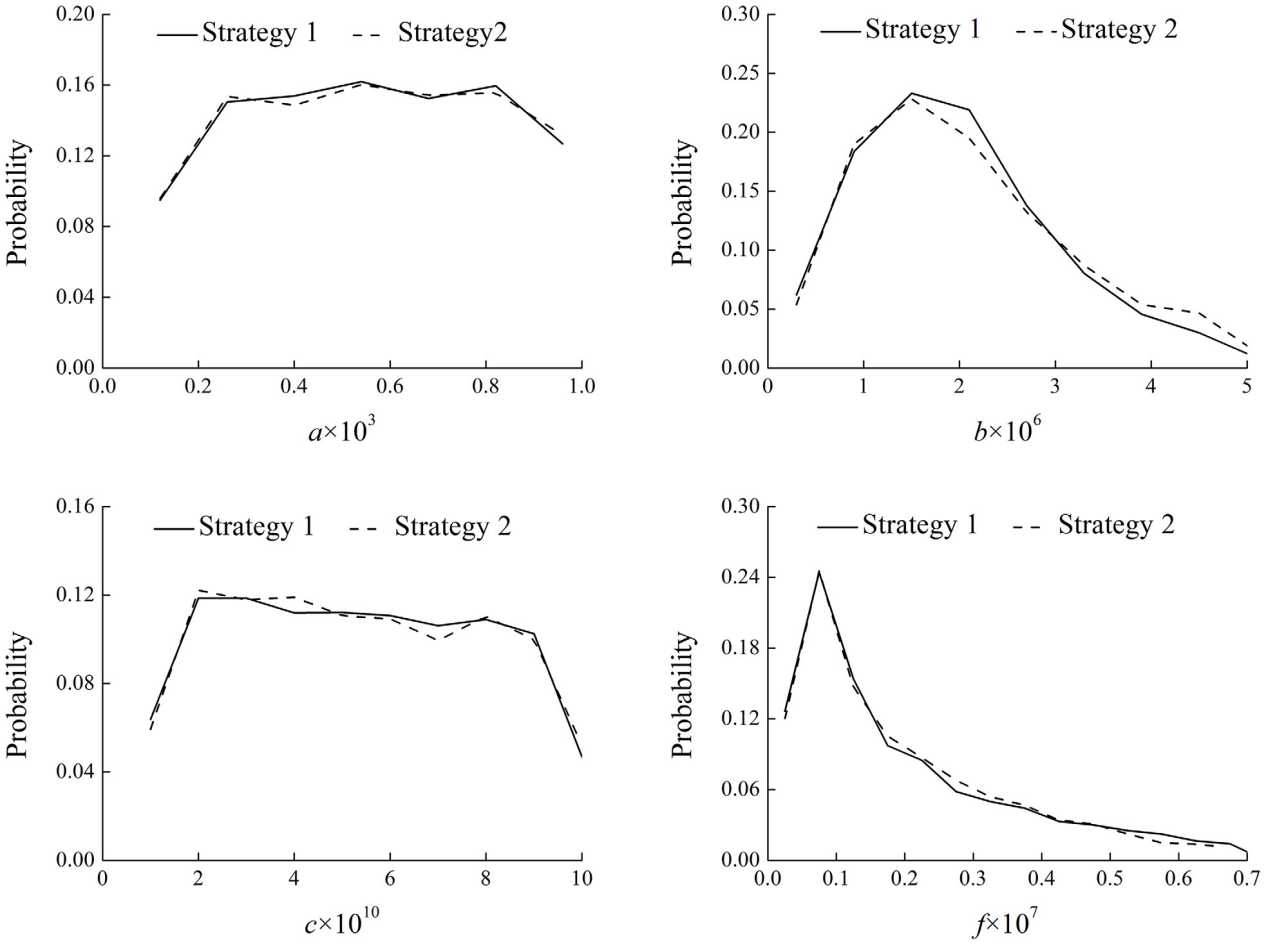
Posterior probability distributions of the behavior-giving parameters with Strategy 1 and 2.

RSA results indicated that the four model parameters, i.e. *a*, *b*, *c* and *f*, were all sensitive parameters and therefore identifiable with both Strategy 1 and Strategy 2 when the UF model was applied to the Qinghe WRP. Fig 3 shows the PPDs of the four parameters from the behavior-giving set for these two strategies, and their curves generally matched each other quite well with only slightly difference. Among the four parameters, *b* and *f* were the most sensitive and identifiable parameters, which was revealed by the remarkable peaks in their PPDs in Fig 3. Indicated by the standard deviation, the uncertainties of *b* and *f* were significantly reduced by 27% and 18% respectively with Strategy 1 and 22% and 22% respectively with Strategy 2. These results were similar to the previous study [17], although the reduction of model parameter uncertainties was not as significant, possibly because of the complicated field conditions and the incomplete monitoring data in the Qinghe WRP as compared with the PSEs. Given that all the four model parameters were sensitive and the most sensitive parameters were well identified with uncertainties significantly reduced, the model could provide robust and reliable predictions for the UF process in the Qinghe WRP.

### Identified Sampling Times

**Fig 4:**
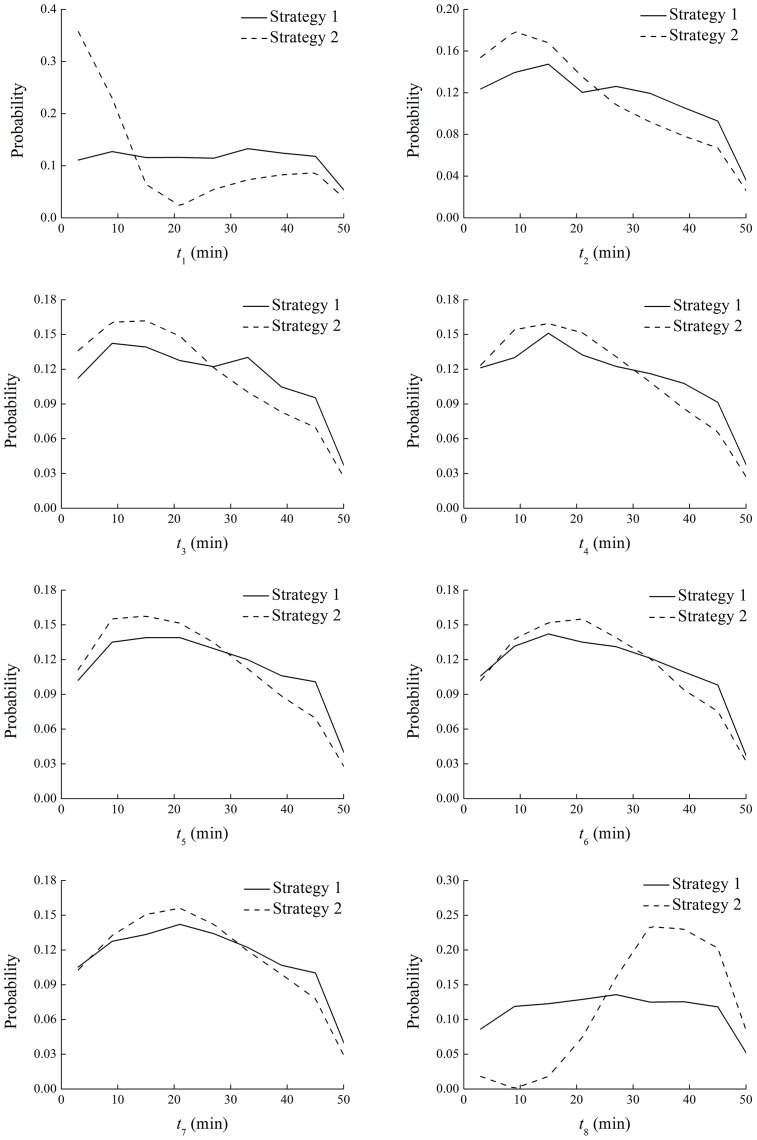
Posterior probability distributions of the behavior-giving *t*_1_~*t*_8_ with Strategy 1 and 2.

The missing sampling times of the observed data in the Qinghe WRP were identified as unknown “parameters”, together with the four model parameters, with both Strategy 1 and Strategy 2. RSA results indicated that the eight sampling times, i.e. *t*_1_~*t*_8_, were all sensitive parameters and therefore identifiable with both strategies. This was reasonable because sampling times were critical model input for the UF model. Fig 4 illustrates the PPDs of the behavior-giving *t*_1_~*t*_8_ with Strategy 1 and Strategy 2. As shown in the figure, the curves for the two strategies separated clearly, and Strategy 2 usually produced more prominent peaks than Strategy 1 in the PPDs of the sampling times. However, except *t*_1_ and *t*_8_, the peaks in the PPDs of the sampling times identified by the two strategies generally coincided with each other, which might suggest the reliability of the identified sampling times by both strategies. For *t*_1_ and *t*_8_, when the model performance was relatively poor as shown in Fig 2, Strategy 2 provided better identification results than Strategy 1. In Fig 4, Strategy 1 gave a “plateau” in the PPD between 10 min and 45 min for both *t*_1_ and *t*_8_, whereas Strategy 2 identified a peak toward the start of the operation cycle for *t*_1_ and a peak toward the end for *t*_8_. Furthermore, judged by the standard deviation, the uncertainties of the identified sampling times were generally lower with Strategy 2. For example, the uncertainty of *t*_8_ with Strategy 2 was reduced by 31% as compared with Strategy 1.

### Comparison of the Two Strategies

Through the results above, it can be found that the two strategies herein proposed for model identification did not make much difference to the identification of four model parameters (see Fig 3). However, Strategy 2 could help better identify the missing model input, i.e. the sampling times (see Fig 4), than Strategy 1 and therefore could give slightly better model performance (see Fig 2). The fundamental difference between these two strategies is the stricter definition of system behavior at the stage of model input identification in Strategy 2. It could be argued that, regardless of computation efficiency, Strategy 1 would produce approximately the same identified model parameters and input as Strategy 2 if it also adopts the stricter definition during Step 2 shown in Fig 1(a). Nevertheless, computation does count in this case. The reason why Strategy 1, as well as the stage of model parameter identification in Strategy 2, used a less stringent definition is that only with such a definition could a reasonable success rate (around 10~15%) of behavior-giving simulations be achieved. To this end, the advantage of Strategy 2 is that, through separating the identification of model parameters from that of sampling times at different stages, it reduced the dimension of the identification problem, increased computation efficiency, and thus facilitated the adoption of stringent model performance requirements. In a more general sense, this study would suggest a dimension-reducing strategy for model identification to separate parameters or input of global impact from those only with local impact, especially under the circumstances of poor data availability.

## Conclusions

A previously developed UF model for the prediction of organics rejection was applied to the UF process in a full-scale WRP. Despite the sparse and incomplete field monitoring data from the Qinghe WRP, encouraging results were still obtained through two specific strategies for this model test study. The two strategies were designed both following a RSA approach, and the missing model input, i.e. sampling times, were identified as unknown “parameters”, together with the four model parameters, with the HSY algorithm based on a LHS approach. The difference between the two strategies was that Strategy 1 aimed to identify the model parameters and sampling times simultaneously, while Strategy 2 tried to separate these two processes to reduce the dimension of the identification problem through an iteration procedure.

The two strategies provided similar results of model performance, and the absolute relative errors between the simulated and observed TOC were generally below 10%. However, Strategy 2 outperformed Strategy 1 for water samples that the model could not simulate well. The four model parameters were all sensitive and identifiable, and the two most sensitive parameters were well identified with uncertainties significantly reduced. Regarding the sampling times, Strategy 2 provided better results than Strategy 1, but they generally agreed with each other on their identified probability distributions. Therefore, at this preliminary stage, it could be inferred that the previously developed UF model could provide robust and reliable predictions for the UF process in the Qinghe WRP. Nevertheless, a well-designed filed monitoring program, including consecutive water sampling within several complete operation cycles of the UF system and full record of sampling times and TOC concentrations in both the feed and permeate, is needed to further test the model.

## Supporting Information

S1 Table Monitoring data of total organic carbon (TOC) of the ultrafiltration process in the Qinghe Wastewater Reclamation Plant. (DOCX)
