# Supplementary material for: Parameter Identification of an Ultrafiltration Model for Organics Removal in a Full-Scale Wastewater Reclamation Plant with Sparse and Incomplete Monitoring Data
Source: PLoS One. 2016 Aug 16;11(8):e0161300. doi: 10.1371/journal.pone.0161300 (PMC4987005; doi:10.1371/journal.pone.0161300)
Supplement: S1 Table — (DOCX) [file pone.0161300.s001.docx]

**S1 Table. Monitoring data of total organic carbon (TOC) of the ultrafiltration process in the Qinghe Wastewater Reclamation Plant.**

| Year/Month | TOC in feed (mg/L) | TOC in permeate (mg/L) |
| --- | --- | --- |
| 2012/06 | 8.45 | 7.29 |
| 2012/07 | 15.19 | 10.15 |
| 2012/08 | 5.88 | 7.09 |
| 2012/09 | 5.25 | 5.03 |
| 2012/10 | 5.77 | 5.12 |
| 2012/12 | 9.82 | 8.16 |
| 2013/01 | 11.32 | 10.18 |
| 2013/03 | 18.65 | 17.31 |
